# A novel regulatory circuit between p53 and GFI1 controls induction of apoptosis in T cells

**DOI:** 10.1038/s41598-019-41684-2

**Published:** 2019-04-19

**Authors:** Charles Vadnais, Riyan Chen, Jennifer Fraszczak, Pierre-Jacques Hamard, James J. Manfredi, Tarik Möröy

**Affiliations:** 1 Institut de recherches cliniques de Montréal (IRCM), Montréal, QC Canada; 2 Sylvester Comprehensive Cancer Center, University of Miami, Miami, FL USA; 3 Icahn School of Medicine at Mount Sinai, New York, NY USA; 4 Département de microbiologie, infectiologie et immunologie, Université de Montréal, Montréal, Canada; 5 Division of Experimental Medicine, McGill University, Montréal, QC Canada

**Keywords:** Tumour-suppressor proteins, Cell death, Post-translational modifications

## Abstract

Here we demonstrate a mode of reciprocal regulation between GFI1 and p53 that controls the induction of apoptosis in T cells. We show that GFI1 prevents induction of p53 dependent apoptosis by recruiting LSD1 to p53, which leads to the demethylation of its C-terminal domain. This is accompanied by a decrease of the acetylation of lysine 117 within the core domain of the murine p53 protein, which is required for transcriptional induction of apoptosis. Our results support a model in which the effect of GFI1’s regulation of methylation at the c-terminus of p53 is ultimately mediated through control of acetylation at lysine 117 of p53. We propose that GFI1 acts prior to the occurrence of DNA damage to affect the post-translational modification state and limit the subsequent activation of p53. Once activated, p53 then transcriptionally activates GFI1, presumably in order to re-establish the homeostatic balance of p53 activity. These findings have implications for the activity level of p53 in various disease contexts where levels of GFI1 are either increased or decreased.

## Introduction

GFI1 is a protein mainly known as a transcription factor involved in hematopoietic cell differentiation by repressing key target genes in multi-potent precursor cells in order to direct their lineage commitment. GFI1 accomplishes this by recruiting the de-methylase LSD1 and histone de-acetylases such as HDAC1 to target promoters^1–5^. GFI1 also has oncogenic activity by promoting survival in leukemic T cells and is required for the development and maintenance of T cell leukemia^6–8^. In this role, GFI1 antagonizes the p53-dependent induction of apoptosis, in part through regulating post-translational modifications at the C-terminal domain of the p53 protein, and as a consequence through transcriptional regulation of the key apoptotic targets *Bax*, *Bbc3* (*Puma*) and *Pmaip1* (*Noxa*)^8^. In addition, GFI1 has been shown to promote DNA repair itself by regulating the activity of DNA repair proteins such as MRE11 and 53BP1 following damage and to contribute to T cell survival in this manner^9^. GFI1 has also been shown to favour the survival and accumulation of myeloid cells in myelo-proliferative disease^10^, potentially due to its role in repressing p53 activity.

It has long been established that the activity of p53 is regulated by the modulation of the protein’s stability by ubiquitination^11–13^. However, in recent years, additional post-translational modifications (PTMs) including acetylation and methylation have emerged as critical factors in the regulation of p53 activity. Transcriptional regulation by p53 of targets involved in specific biological functions has been associated with sets of corresponding PTMs. For instance, mutant mouse p53 proteins carrying lysine to arginine mutations at 3 lysine residues (117, 161, 162, corresponding to residues 120 and 164 in the human protein, which has one fewer lysine at this location) in the DNA-binding domain of p53 are deficient in inducing apoptosis, cell cycle arrest and senescence, whereas a p53 protein carrying a single lysine to arginine substitution at residue 117 (120 in the human protein) is only deficient in inducing apoptosis^14^.

Regulation of p53 through modification of its C-terminal Domain (CTD) has also been shown to be important for its activity, however its exact role is not clearly defined, as there is conflicting evidence in the literature. Studies have shown that the CTD is involved in the regulation of p53 DNA binding, the interaction with its negative regulator MDM2, and the regulation of its stability, among other functions^15–18^. Of particular interest here are several reports showing that the impact of the CTD on p53 activities is likely context- and cell type-specific. For example, through ectopic expression of mutant p53 proteins in several human cancer cell lines, it was found that p53 binding to the key apoptotic target *Puma* requires the CTD^19^, while later work using mouse models and murine primary cells showed that expression of CTD-deleted p53 actually increased *Puma* expression and induction of cell death in thymocytes^20^. Given the complexity and the number of potential residue modifications within the p53 protein, their individual roles are not yet fully understood, and neither is the interplay between modifications of different residues.

Here, we focus on detailing how GFI1 activity affects post-translational modification of p53 at both the C-terminus and at lysine 117, and how this translates into changes in induction of apoptosis in T cells. We take advantage of multiple mouse models to characterize the mechanism by which GFI1 regulates p53 activity in this way. We first use a *Gfi1* KO mouse model as well as a model expressing a mutant GFI1 protein with a proline to alanine mutation at residue 2 (P2A), which affects its interaction with other proteins, notably LSD1^5,21^. Our *Gfi1* KO model features a GFP coding sequence, which is inserted in-frame with the initiation codon of *Gfi1* and replaces exons 3–5 of the *Gfi1* gene, resulting in the production of a GFP transcript under the control of *Gfi1*’s regulatory sequences and the absence of GFI1 protein^22^.

We also use a mouse model expressing a truncated form of the p53 protein lacking the 24 C-terminal amino acids^20^. This model (p53 ΔCTD) lacks several lysine residues (K370, K372, K373, K381, K382, K386 K387 in the mouse protein, corresponding to K370, K372, K373, K381, K382, K386 in the human protein) that can be modified by methylation and acetylation. Finally, we use a knock-in mouse expressing a mutant p53 in which a lysine to arginine substitution at residue 117 (p53 K117R) prevents an acetylation event necessary for the transactivation by p53 of genes critical for the induction of apoptosis^14^.

Here, we show that in T cells, GFI1 blocks the accumulation of methylation marks at the CTD of p53, which in turn inhibits the acetylation of K117. In addition, we demonstrate that GFI1 is activated by p53 following DNA damage. Given that GFI1 antagonizes the function of p53, our results suggest that GFI1 plays a role in post-translational negative feedback regulation of p53 induced by p53’s own transcriptional activity, as is seen with other regulators of p53, such as MDM2.

## Results

### The intermediate domain of GFI1 interacts with the C-terminal domain of p53

**Figure 1:**
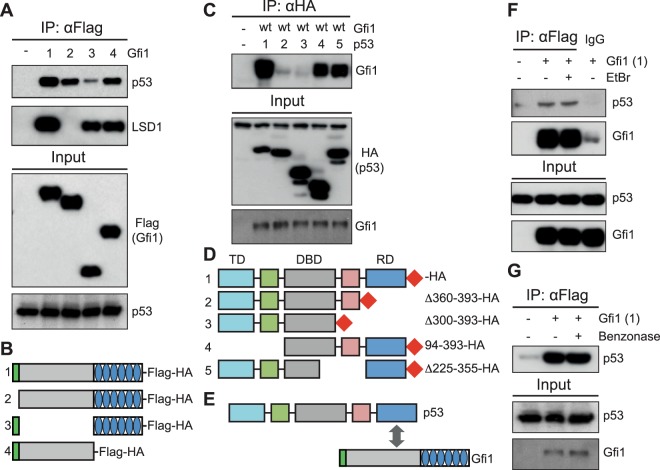
The intermediate domain of GFI1 interacts with the C-terminal domain of p53 (**A**) 293T cells were transfected with vectors expressing variants of the GFI1-Flag-HA fusion protein. Nuclear extracts from these proteins were immunoprecipitated for Flag, separated by SDS-PAGE and blotted for the indicated proteins. Blots from input controls are shown below. (**B**) Schematic representation of the variant GFI1 proteins used in (**A**). (**C**) 293T cells were transfected with vectors expressing variants of the p53-HA fusion protein as well as GFI1-Flag protein without an HA tag. Extracts were immunoprecipitated for HA and blotted for the indicated proteins. (**D**) Schematic representation of the variant p53 proteins used in (**C**). (**E**) Proposed model of the interaction between GFI1 and p53. (**F**) GFI1-Flag-HA fusion protein was immunoprecipitated in 293T cells in the presence or absence of ethidium bromide and blotted for p53. (**G**) GFI1-Flag-HA fusion protein was immunoprecipitated in 293T cells in the presence or absence of benzonase and blotted for p53.

To fully characterize the interaction between GFI1 and p53, we first performed co-immunoprecipitation (Co-IP) experiments using a series of GFI1 mutants. These experiments showed that the full-length form of GFI1 is capable of interacting with p53, and that deletion of the intermediary domain of GFI1 prevents this interaction, whereas deletions of the SNAG domain or of the DNA binding domain of GFI1 had no effect on the binding to p53 (Fig. 1A,B). This is in contrast with the interaction between GFI1 and the de-methylase enzyme LSD1, which requires the SNAG domain of GFI1 for interaction. Further analysis of GFI1 variants with deletions in the intermediary domain showed that different sizeable deletions across the intermediate domain decreased the interaction with p53, without identifying a single critical region, suggesting that the domain as a whole may be involved (Suppl. Fig. 1A). Conversely, Co-IP experiments using deletion variants of the p53 protein showed that its C-terminal domain, but not the N-terminal domain of the protein, is required for its interaction with GFI1 (Fig. 1C,D), suggesting that the intermediate domain of GFI1 interacts with p53’s C-terminal end (Fig. 1E). Interestingly, further Co-IP experiments showed that the interaction between the two proteins is unaffected by the presence of benzonase or ethidium bromide (EtBr), indicating that the p53/GFI1 interaction is independent of DNA binding (Fig. 1F,G, Supplementary Fig. 1B).

### GFI1 antagonizes p53 CTD methylation and K117 acetylation, and restrains the p53-dependent apoptotic response to DNA damage

**Figure 2:**
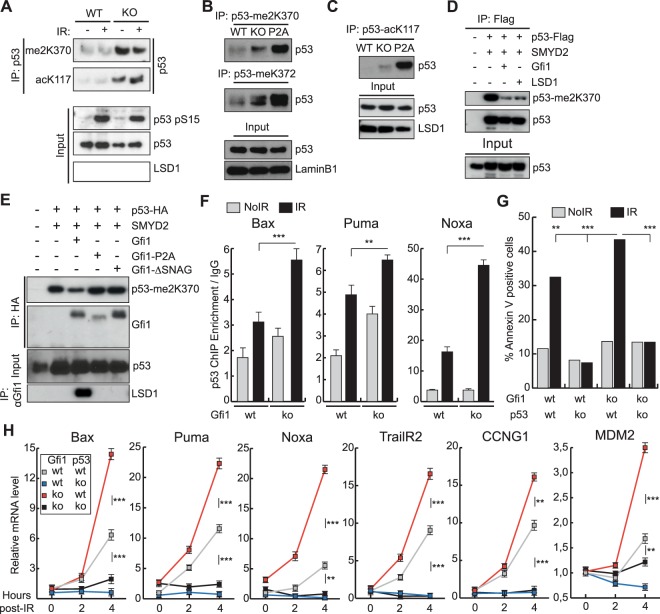
GFI1 modulates the induction of apoptosis by p53 through CTD and core domain PTMs. (**A**) Thymocytes from GFI1 WT and KO mice were exposed to 5Gy IR or left untreated. Nuclear extracts from these cells were immunoprecipitated with p53-me2K370 or p53-acK117 specific antibodies and blotted for total p53. (**B**) Nuclear extracts from GFI1 WT, KO and P2A thymocytes were immunoprecipitated with p53-me2K370 or p53-meK372 specific antibodies and blotted for total p53. (**C**) Nuclear extracts as in B were immunoprecipitated with a p53-acK117 specific antibody and blotted for total p53. (**D**) 293T cells were transfected with the indicated combinations of p53-Flag, SMYD2, GFI1 and LSD1 expression vectors. Nuclear extracts were immunoprecipitated with an anti-Flag antibody and blotted for p53-me2K370 and total p53. (**E**) 293T cells were transfected with the indicated combinations of p53-HA, SMYD2, GFI1, GFI1-P2A and GFI1-ΔSNAG expression vectors. Nuclear extracts were immunoprecipitated with anti-HA or anti-GFI1 antibodies and blotted as indicated. (**F**) Thymocytes were extracted from GFI1 WT and KO mice, exposed to 5Gy IR or left untreated and fixed with formaldehyde after 2 hours. Chromatin immunoprecipitation was performed to isolate p53-bound DNA fragments. Fold enrichment of the indicated target promoters relative to a non-specific IgG control is shown. Error bars represent s.d. *P* values: *=<0.05, **=<0.01, ***=<0.001, calculated from a Welch corrected t-test. (**G**) Thymocytes were extracted from mice carrying combinations of Gfi1 KO and p53 KO. Cells were exposed to 5Gy IR or left untreated and were stained for Annexin V 4 hours later. The proportion of Annexin V positive cells as measured by FACS is shown. Statistical significance was calculated using Fisher’s exact test. (**H**) mRNA was extracted from thymocytes as in (**G**). The levels of the indicated genes were measured at the indicated time points after IR by qPCR relative to *Gapdh*.

In order to measure the methylation status of the p53 CTD, we generated antibodies for p53-me2K370 and p53-me2K372 and confirmed their binding efficacy and specificity *in vitro* (Supplementary Fig. 2A). Using Co-IP experiments with these antibodies and others specific for post-translationally modified p53 residues, we show that, in addition to the previously reported increase in K372 mono-methylation^8^, K370 di-methylation as well as K117 acetylation was also increased in *Gfi1* KO thymocytes. Interestingly, these increases were independent of DNA damage induction by irradiation, unlike the well-described phosphorylation of S15 (Fig. 2A). The increase in K117 acetylation is noteworthy as this PTM is well established as being required for induction of key apoptotic genes downstream of p53. Furthermore, we show that in thymocytes extracted from mice carrying the P2A mutant variant of *Gfi1*^21^, which cannot interact with LSD1^5^, both K372 mono-methylation and K370 di-methylation marks were increased (Fig. 2B), similarly to what was observed in the absence of GFI1. Furthermore, GFI1 P2A expressing cells also displayed increased K117 acetylation (Fig. 2C).

We further tested the ability of GFI1 to block the accumulation of methylation marks at the C-terminal end of p53 by overexpressing p53, SMYD2, and either GFI1 or LSD1 in 293T cells. Overexpression of SMYD2 readily induced K370 di-methylation in these cells, while a catalytically inactive SMYD2-GEE mutant did not, as expected (Suppl. Fig. 2A)^23^. We found that concomitant overexpression of either GFI1 or LSD1 blocked the accumulation of K370 di-methylation induced by SMYD2 (Fig. 2D). This effect was dose-dependent, as overexpression of increasing levels of exogenous GFI1 showed a proportional reduction in K370 di-methylation (Suppl. Fig. 2B). Importantly, overexpression of mutant variants of GFI1 that cannot interact with LSD1, namely GFI1 P2A and GFI1 ΔSNAG, did not lead to a decrease in p53-K370 di-methylation (Fig. 2E). Together, these data demonstrate that GFI1 causes de-methylation of the C-terminal domain of p53 through recruitment of LSD1, as previously suggested^8^. Moreover, our results show that GFI1 antagonizes the accumulation of the acK117 mark, which is key to inducing apoptosis. As expected, the increase in C-terminal methylation and K117 acetylation in the *Gfi1* KO context correlated with a greater induction of the pro-apoptotic p53 target genes *Bax*, *Puma*, *Noxa* and *TrailR2* in response to IR exposure, as compared to WT cells (Suppl. Fig. 2C). This was likely due to increased binding of p53 to the promoter of these genes, as assessed by chromatin immunoprecipitation (Fig. 2F).

To confirm that the increased apoptotic response we observed in the *Gfi1* KO cells was p53 dependent we crossed our *Gfi1* KO mice with p53 KO mice. We found that the increased apoptotic response in *Gfi1* KO cells was no longer observed in the context of a p53 KO, i.e. when p53 was also absent (Fig. 2G). Accordingly, the greater induction of p53 targets *Bax*, *Puma*, *Noxa* and *TrailR2* following IR exposure in *Gfi1* KO cells as compared to WT cells, was completely eliminated in Gfi1/p53 double KO cells (Fig. 2H). Interestingly, the induction of the negative feedback regulators of p53, *Mdm2* and *Ccng1* was also found to be increased in *Gfi1* KO cells, with this effect once again being dependent on p53 itself (Fig. 2H). Taken together, these data support a model whereby GFI1 antagonizes p53 CTD methylation and K117 acetylation, which leads to a restrained p53-dependent apoptotic response to DNA damage.

### GFI1’s impact on p53 activity is ultimately mediated through changes in K117 acetylation

**Figure 3:**
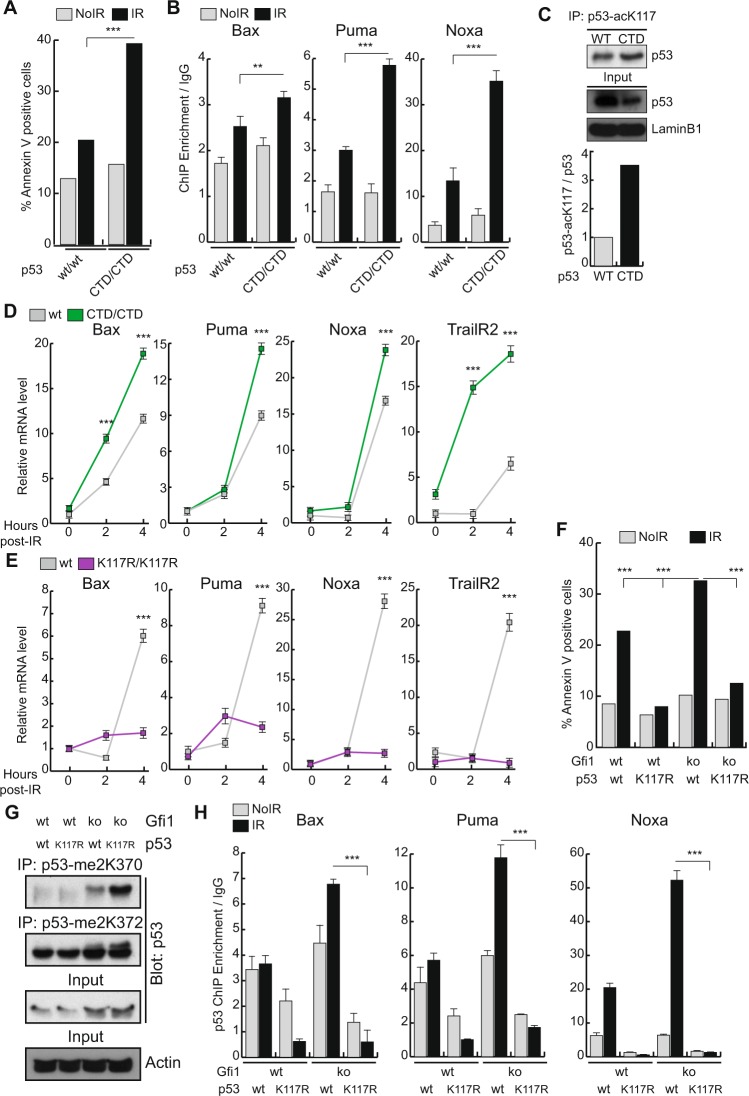
The effect of GFI1 in p53 regulation is mediated through K117 of p53. (**A**) Thymocytes were extracted from p53 WT and p53 ΔCTD mice. Cells were exposed to 5Gy IR or left untreated and were stained for Annexin V 4 hours later. The proportion of Annexin V positive cells as measured by FACS is shown. (**B**) Thymocytes were extracted from p53 WT and p53 ΔCTD mice, exposed to 5 Gy IR or left untreated and fixed with formaldehyde after 2 hours. Chromatin immunoprecipitation was performed to isolate p53-bound DNA fragments. Fold enrichment of the indicated promoter targets relative to a non-specific IgG control is shown. (**C**) Nuclear extracts from p53 wt and p53 CTD thymocytes were immunoprecipitated with a p53-acK117 specific antibody and blotted for total p53. (**D**) mRNA was extracted from thymocytes as in (**A)**. The levels of the indicated genes were measured at the indicated time points after IR by qPCR relative to *Gapdh*. (**E**) mRNA was extracted from the thymocytes of p53 WT and p53 K117R mutant mice. The levels of the indicated genes were measured at the indicated time points after IR by qPCR relative to *Gapdh*. (**F**) Thymocytes were extracted from mice carrying combinations of GFI1 KO and p53 K117R mutation. Cells were exposed to 5Gy IR or left untreated and were stained for Annexin V 4 hours later. The proportion of Annexin V positive cells as measured by FACS is shown. (**G**) Thymocytes from mice carrying combinations of GFI1 KO and p53 K117R mutation were exposed to 5Gy IR or left un-irradiated. Nuclear extracts from these cells were immunoprecipitated with p53-me2K370 or p53-me2K372 specific antibodies and blotted for total p53. (**H**) Thymocytes were extracted from WT, p53 K117R, GFI1 KO and p53 K117R GFI1 KO mice, exposed to 5Gy IR or left untreated and fixed with formaldehyde after 2 hours. Chromatin immunoprecipitation was performed to isolate p53-bound DNA fragments. Fold enrichment of the indicated promoter targets relative to a non-specific IgG control is shown.

In order to better understand how the GFI1-dependent regulation of p53 PTMs affects the induction of apoptosis, we used a mouse model expressing a variant of p53 lacking its C-terminal domain^20^. Similarly to what we observed in the *Gfi1* KO model, the absence of p53 CTD leads to increased apoptosis in thymocytes exposed to IR (Fig. 3A), as well as increased recruitment of p53 to the promoters of *Bax*, *Puma*, *Noxa* (Fig. 3B), an increase in the level of p53 K117 acetylation (Fig. 3C) and a greater induction of the expression of apoptotic genes following IR (Fig. 3D). These results also suggest that a p53 protein carrying an un-methylated CTD, as mediated by the activity of GFI1, is less able to induce the apoptotic response than the methylated one and that the complete removal of the CTD prevents this check on p53 activity, leading to unrestrained p53 activation, as is seen in Gfi1 KO cells.

To confirm this hypothesis, we then used another knock-in mouse model expressing a mutant p53 with a lysine to arginine substitution at amino acid 117 (p53 K117R)^14^. As expected, thymocytes extracted from these mice did not show activation of the pro-apoptotic genes *Bax*, *Puma*, *Noxa* and *TrailR2* following IR exposure (Fig. 3E). We crossed the p53 K117R line with our GFI1 KO line and found that the increase in apoptosis following IR observed in GFI1 KO cells was completely eliminated in cells that also carried the p53 K117R mutation (Fig. 3F). Most importantly, we still detected an increase in p53 C-terminal K370 di-methylation and K372 di-methylation in the GFI1 KO p53 K117R cells after IR, similar to what we observed in GFI1 KO cells (Fig. 3G). This shows that the regulation exerted at the C-Terminal domain of p53 is not sufficient to induce apoptosis on its own and strongly suggests that the effect of GFI1 on apoptosis is ultimately mediated through the regulation of p53 K117 acetylation. This is supported by p53 ChIP experiments using combinatorial GFI1 KO and p53 K117R mutant mice, which show that while GFI1 KO leads to an increase in p53 recruitment to promoters of the apoptotic genes *Bax*, *Puma* and *Noxa*, it does not lead to any increase in the recruitment of p53K117R to these promoters (Fig. 3H). This also suggests that the increase in C-terminal methylation of p53 in GFI1 KO cells only leads to an increase in apoptotic response after acetylation at K117.

### GFI1 and p53 are part of a regulatory feedback loop

**Figure 4:**
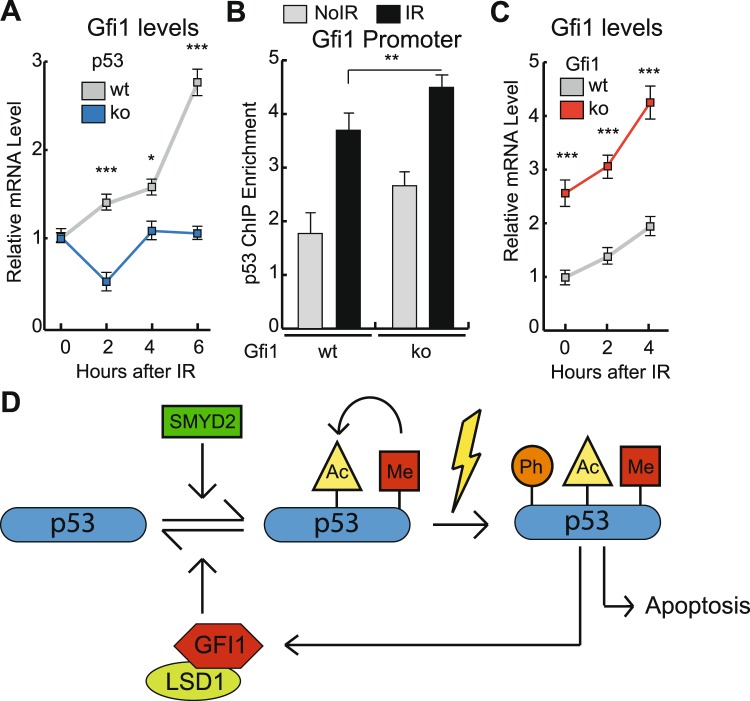
GFI1 is a negative feedback regulator of p53 (**A**) Thymocytes were extracted from p53 WT or KO mice. Cells were exposed to 5Gy IR or left untreated. mRNA was extracted and the levels of *Gfi1* were measured at the indicated time points by qPCR relative to *Gapdh*. (**B**) Thymocytes were extracted from GFI1 WT and KO mice. Cells were exposed to 5Gy IR or left untreated and fixed with formaldehyde after 2 hours. Chromatin immunoprecipitation was performed to isolate p53-bound DNA fragments. Fold enrichment amplification at the *Gfi1* promoter relative to a non-specific IgG control is shown. (**C**) mRNA was extracted from thymocytes as in (**B)**. The levels of KO mutant mRNA were measured at the indicated time points after IR by qPCR relative to *Gapdh*. (**D**) Diagram representing the regulatory circuit between GFI1 and p53.

The data presented here, as well as previous reports, show that GFI1 is a critical regulator of p53 activities, at least in thymocytes^8^. Other studies indicated that p53 might regulate GFI1 expression, suggesting a possible regulatory feedback loop between these two proteins, in a manner similar to the well-established p53-MDM2 negative feedback loop. To determine whether p53 controls GFI1 in thymocytes, we measured the mRNA levels of *Gfi1* in p53 WT and KO cells at baseline as well as following exposure to IR and observed that while *Gfi1* mRNA levels were not altered by p53 KO at baseline, they increased following exposure to IR in a p53 dependent manner (Fig. 4A). Consistent with this, ChIP enrichment of p53 at the Gfi1 promoter increased following IR exposure (Fig. 4B). In order to further study the relationship between GFI1 and p53, we took advantage of the fact that the Gfi1 KO construct expresses a non-functional GFI1-GFP mutant mRNA that can still be measured using primers that recognize the 3’UTR of Gfi1^22^. Interestingly, in cells expressing the Gfi1-GFP mRNA (and thus lacking a functional GFI1 protein), the binding of p53 to the Gfi1 promoter was increased both in the absence of DNA damage as well as following exposure to IR (Fig. 4B). Furthermore, the levels of the GFI1-GFP mRNA were higher than the baseline levels of normal Gfi1 mRNA and these levels increased even further following irradiation (Fig. 4C). These results are consistent with a model whereby GFI1 is a p53-activated negative feedback regulator of p53, since in the absence of a functional GFI1 protein p53 binds more strongly to the promoter of GFI1 and induces it without being repressed in return.

Our data support a model describing a novel regulatory loop between GFI1 and p53, which controls p53 activity in T cells. While methyltransferases- and acetylases-dependent PTMs prime p53 for potent pro-apoptotic responses after DNA damage, GFI1 favours the removal of these marks, through the recruitment of the lysine de-methylase LSD1 at K370 and K372, and through an unknown mechanism at K117, which leads to restrained p53 activities. Upon exposure to DNA damage, the modified form of p53 is more strongly activated than its unmodified counterpart and it promotes both a potent apoptotic response and the transactivation of GFI1, which in turn leads to a de-methylation of p53 and its inactivation hence forming a novel negative feedback loop (Fig. 4D).

## Discussion

Our results indicate that a regulatory circuit exists between GFI1 and p53 that controls the induction of apoptosis in T cells. We speculate that this link may exist in other cell types that express GFI1, such as myeloid cells, where a link between the two proteins has also been suggested^10^.

Our study builds on a previously suggested mechanism of regulation of p53 activity by GFI1, which proposed that the two proteins form a complex with the lysine de-methylase LSD1. Using deletion mutants and biochemical approaches we were able to characterize the domains critical for the interaction between GFI1 and p53 proteins, i.e. the intermediate domain of GFI1 and the C-Terminal domain of p53. Interestingly, the intermediate domain of GFI1 has also recently been shown to mediate its interaction with the DNA repair protein MRE11 and the arginine methyltransferase PRMT1 that targets it^9^. This suggests that this domain of GFI1 is involved in the regulation of functions related to p53 activity and DNA repair, whereas the SNAG domain at the very N-terminal end of GFI1 is more closely, but not exclusively, associated with its transcriptional activity. This also indicates that GFI1 has two very different mechanisms of action, one as a DNA binding transcription factor and another one as a bridging factor facilitating contact between enzymes and their protein substrates and enabling specific PTMs to take place.

We provide new evidence that the recruitment of LSD1 to p53 is necessary for the effect of GFI1 on p53 CTD methylation and consequently on apoptosis. Furthermore, our work with the p53ΔCTD mouse model shows that this form of p53 is overactive and induces apoptosis more readily than wild-type p53. This is in agreement with the original report describing this mouse model and defines the CTD as a negative regulator of p53 activity in thymocytes^20^. These data also suggest that a non-methylated CTD, as seen in GFI1 expressing cells, acts in a way that represses p53 activation.

Importantly, we show that C-terminal methylation on its own is insufficient to control p53-induced apoptosis but also requires acetylation at lysine 117 to have an effect of p53 activity. Our experiment with a K117R mutant variant of p53 demonstrates this, since cells from this mutant do not undergo apoptosis regardless whether Gfi1 is present or not. This mutation effectively renders the increase in C-terminal domain methylation seen in Gfi1 KO cells inoperative or incapable of increasing p53’s activity. These results support the notion that GFI1’s effect on programmed cell death is ultimately dependent on its ability to control acetylation of the K117 residue of p53 *via* the regulation of CTD methylation. It also suggests that other regulatory pathways that involve methylation events at p53’s CTD may ultimately be mediated through PTM of other p53 domains. Notably, this idea is consistent with findings indicating that the CTD of p53 may be involved in conformational changes of the DNA binding domain, which contains the K117 residue^24^.

All effects of GFI1 on post-translational modifications of p53 that we analyzed here are independent of both exposure to irradiation and interaction with DNA, suggesting that GFI1’s role is to control the extent to which p53 is “primed” to induce apoptosis once DNA damage occurs. In the context of GFI1 KO, there appears to be excessive priming of p53, leading to greater induction of apoptosis, although a death-inducing stimulus, such as exposure to IR, is still required to fully activate p53. Although this function of GFI1 is distinct from its canonical role as a DNA binding transcription factor, it remains to be determined whether GFI1 additionally plays a direct transcriptional role in regulating the induction of apoptosis and to which extent both functions overlap.

Although data exist in the literature showing that p53 can regulate GFI1, other conflicting evidence suggests that this regulation is context specific. For instance, p53 KO BM Lineage^-^Sca^+^c-Kit^+^ progenitor cells have been shown to have lower baseline levels of GFI1^25^, while another group has shown this effect occurs in the spleen and thymus, but not in the bone marrow^20^. However, others have shown that p53 represses GFI1 expression specifically following DNA damage^26^. This is in contrast with the data presented here showing that GFI1 is transcriptionally activated following IR exposure in a p53-dependent manner. This discrepancy may be explained by the different cell systems used, i.e. murine primary T cells for the present study versus a murine pro B cell line and several human cancer cell lines in the previous study^26^. Moreover, our own experiments were carried out within a much shorter time frame after exposure to DNA damage (2–6 h vs. >24 h). Nevertheless, our data otherwise agree with findings from the literature that GFI1 antagonizes the p53-dependent apoptotic response. Given the data present here and the fact that the activation of key pro-apoptotic targets of p53 is dampened by the action of GFI1, it is likely that GFI1 is a negative feedback regulator of p53 in T cells. Indeed, the increased binding of p53 at the *Gfi1* promoter, and the increased *Gfi1* transcript levels in the absence of a functional GFI1 protein strongly support this notion and a model in which p53 and GFI1 are members of a regulatory circuit controlling apoptosis in T-cells.

## Methods

### Mouse Strains

Gfi1 KO and Gfi1 P2A mice used in this study have been previously described^5,10,27,28^. p53CTD Mutant Mice^20^ and p53 K117R^14^ mutant mice were graciously provided by the laboratories of James Manfredi and Wei Gu, respectively. Mice have been bred on to C57BL/6 genetic background and were maintained in a Specific-Pathogen-Free Plus environment at the Institut de recherches cliniques de Montreal (IRCM). The Institutional Review Board of the IRCM approved all animal protocols and experimental procedures were performed in compliance with IRCM and CCAC (Canadian Council of Animal Care) guidelines.

### Cell Culture

293T cells were maintained in RPMI media (Multicell) supplemented with 10% Bovine Growth Serum (RMBIO Fetalgro) and 100 IU Penicillin and 100 μg/ml Streptomycin (Multicell). We verified that none of the cell lines used in this study were found in the Register of Misidentified Cell Lines maintained by the International Cell Line Authentication Committee (http://iclac.org/databases/cross-contaminations/).

### Annexin V Staining

Cells were extracted from mouse thymi and treated with red blood cell lysis buffer for 5 min at RT. Cells were then resuspended in RPMI media as for cell culture. Cells were exposed to 5 Gy IR and incubated for 4 hours prior to staining using the FITC Annexin V Apoptosis Kit I (BD Pharmingen) according to the manufacturer’s instruction. Cell staining was measured on a FACSCalibur.

### Antibodies

For the detection of p53-me2K370 and p53-me2K372, polyconal antibodies were generated in rabbits against the peptide sequences CSSHLK(me2)SKKGQS and CSHLKSK(me2)KGQST. The following commercial antibodies were used: LaminB1 ab16048 (Abcam), p53 1C12 (Cell Signaling Technology), p53-meK372 ab16033 (Abcam), GFI1 AF3540 (R&D Systems), GFI1 H-200 (Santa-Cruz), LSD1 EPR6826 (Epitomics 5839-1), HA 12CA5 (Sigma), Flag M2 (Sigma).

### Co-Immunoprecipitation

For each Immunoprecipitation, 10 million cells were lysed in buffer I (0.5% NP-40, 10 mM Hepes, 10 mM KCl, 2 mM EDTA, 10% Glycerol, Complete protease inhibitor (Roche), pH7.5), incubated on ice for 10 min and centrifuged for 10 min at 13,000 rpm. Pellets were lysed in 500 μl buffer II (50 mM Sodium Phosphate, 300 mM NaCl, 1 mM β-mercaptoethanol, 10% Glycerol, 0.5% NP-40, 0.5% Triton X-100, Complete protease inhibitor (Roche), pH 7.5), mixed by vortexing and sonicated twice on a Brason digital sonifier for 10 sec at 50% output followed by 10 min incubation on ice and centrifuged for 10 min at 13,000 rpm. Supernatant was incubated for 2 h using the antibody of interest followed by 1 h incubation with Protein-A or -G agarose beads (Roche). Beads were washed 4 times with buffer II and proteins were extracted by boiling the beads for 5 min in SDS-PAGE sample loading buffer prior to separation by SDS-PAGE electrophoresis.

### Expression level measurement

Total mRNA was extracted from cells using the RNeasy Micro Kit (Qiagen) according to the manufacturer’s instructions. mRNA was reverse transcribed to cDNA using superscript II (Thermo Fisher Scientific).

## Supplementary information

Supplementary Figures

## Data Availability

No datasets were generated or analyzed as part of this study.
