# Supplementary material for: A novel regulatory circuit between p53 and GFI1 controls induction of apoptosis in T cells
Source: Sci Rep. 2019 Apr 19;9:6304. doi: 10.1038/s41598-019-41684-2 (PMC6474872; doi:10.1038/s41598-019-41684-2)
Supplement: Supplementary file 1 — Supplementary Figures [file 41598_2019_41684_MOESM1_ESM.pdf]

A novel regulatory circuit between p53 and GFI1 controls induction of apoptosis in T cells

Charles Vadnais, Riyan Chen, Jennifer Fraszczak, Pierre-Jacques Hamard,

James J Manfredi, Tarik Möröy

Supplementary Figures and legends

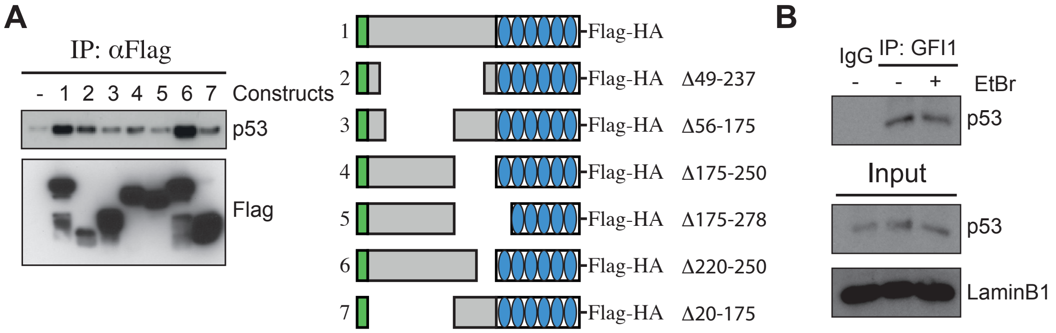

Supplementary Figure 1

A 293T cells were transfected with variants of the GFI1-Flag-HA fusion protein. Extracts were immunoprecipitated for Flag and blotted for the indicated proteins. Schematic representation of the variant p53 proteins is shown on the right.

B Nuclear extracts from thymocytes were immunoprecipitated with an anti GFI1 antibody and blotted for p53.

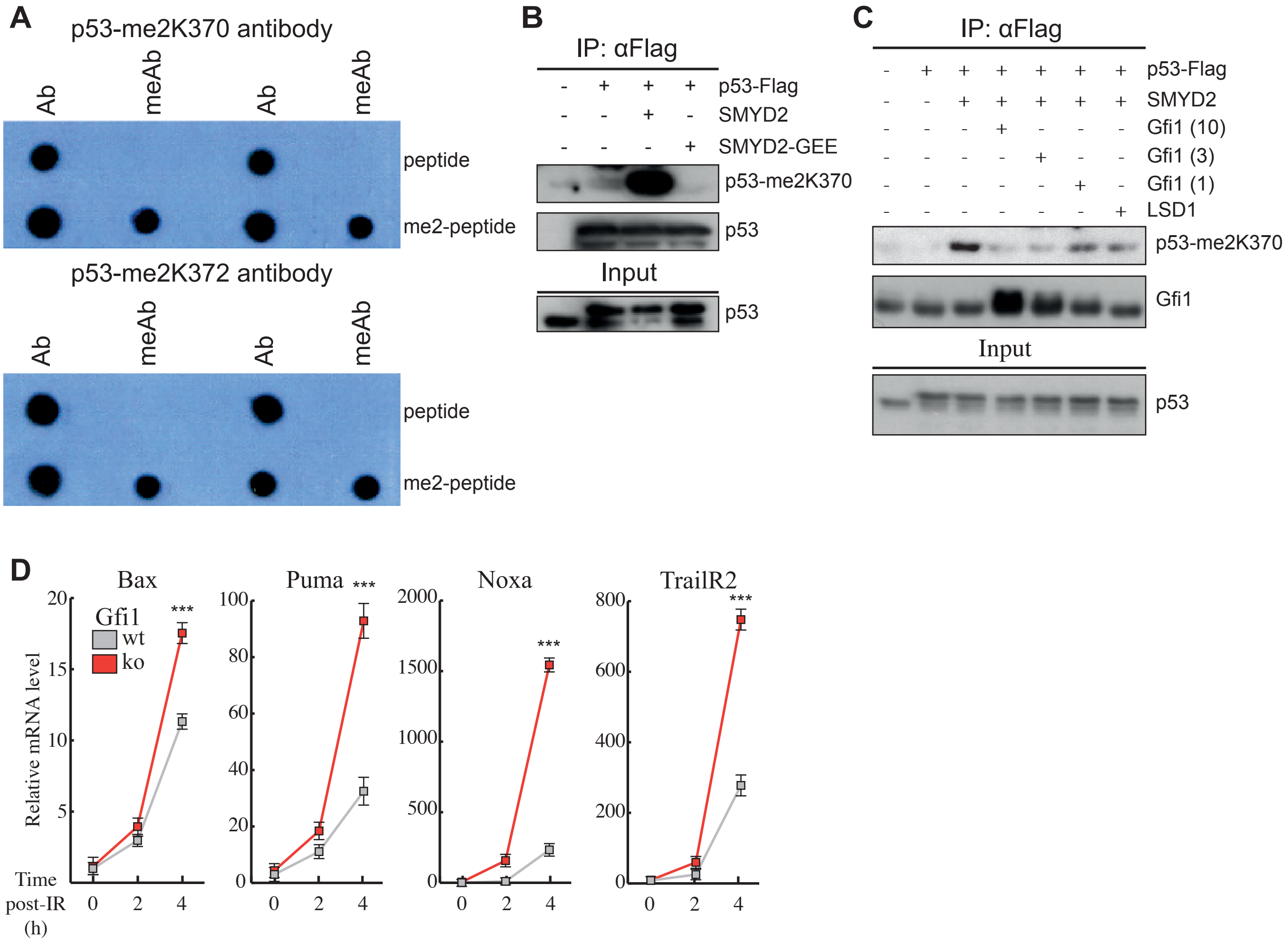

Supplementary Figure 2

A Antibodies against p53-me2K370 peptides and unmethylated peptides were generated. The antibodies were then tested against the unmethylated and methylated forms of the peptides to confirm the specificity of the anti-methyl antibodies for the methylated peptides. Antibodies for p53-me2K372 were generated and tested in the same manner.

B 293T cells were transfected with the indicated combinations of p53-Flag, SMYD2 and SMYD2-GEE expression vectors. Nuclear extracts were immunoprecipitated with an anti Flag antibody and blotted for p53-me2K370 and total p53.

C 293T cells were transfected with the indicated combinations of p53-Flag, SMYD2 and GFI1 expression vectors. Nuclear extracts were immunoprecipitated with an anti Flag antibody and blotted for p53-me2K370 and total p53.

D Thymocytes were extracted from Gfi1 KO mice. Cells were exposed to 5Gy IR and mRNA was extracted from cells. The levels of the indicated genes were measured at the indicated time points after IR by qPCR relative to Gapdh.

Figure 1A

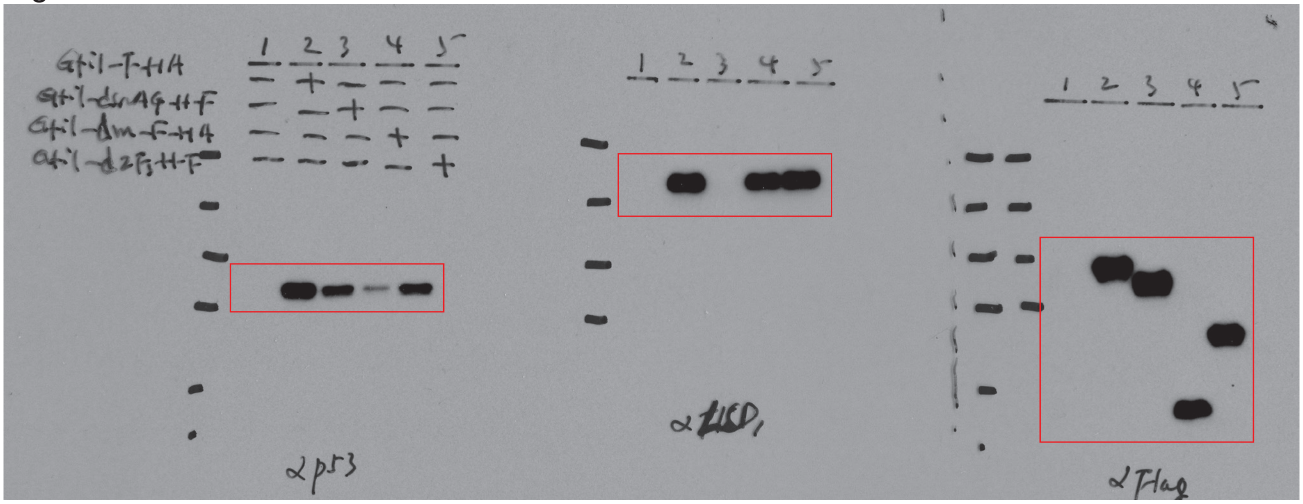

p53 LSD1 Flag

Figure 1C

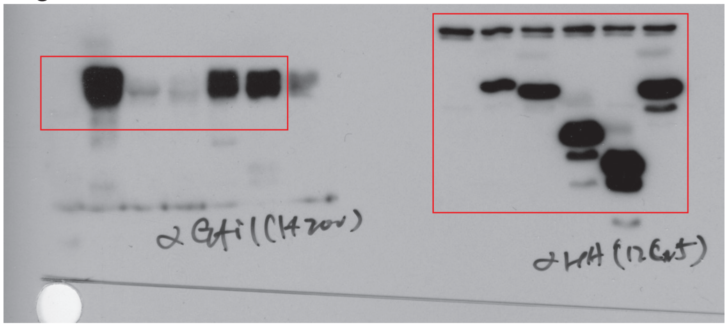

GFI1 HA

Figure 1A | Figure 1G

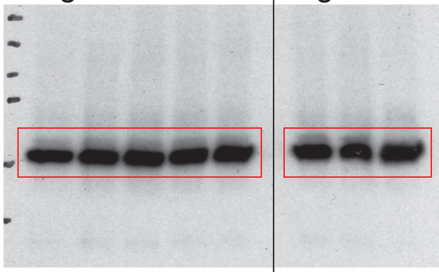

p53 (Input) | p53 (Input)

Figure 1F

IP

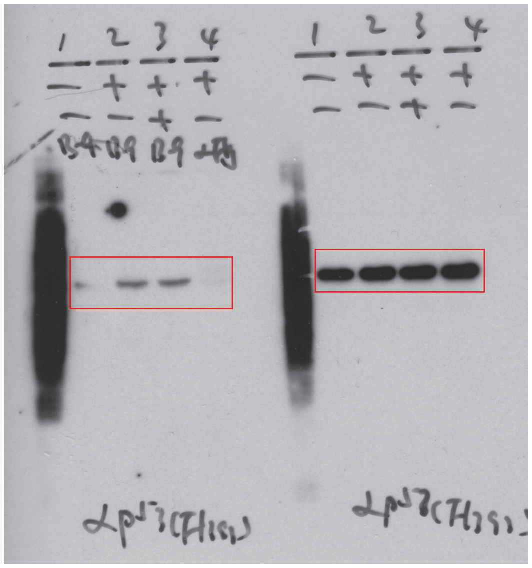

p53 Gfi1

Input

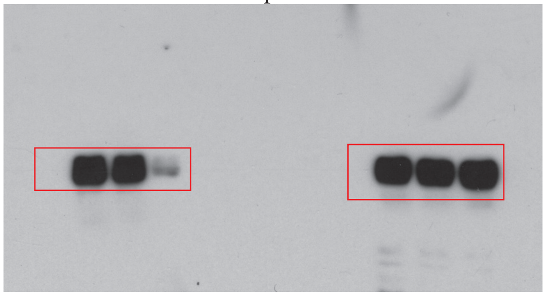

p53 Gfi1

Figure 1G

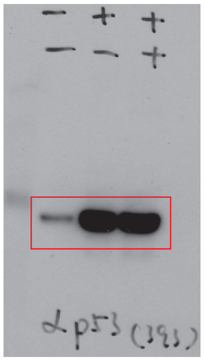

p53

Figure 1C | Figure 1G

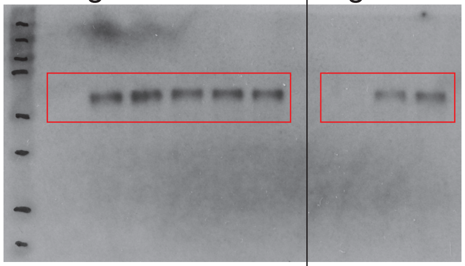

Gfi1 (Input) | Gfi1 (Input)

Supplementary Figure 3

Uncropped images of blots shown in Figure 1.

Figure 2A

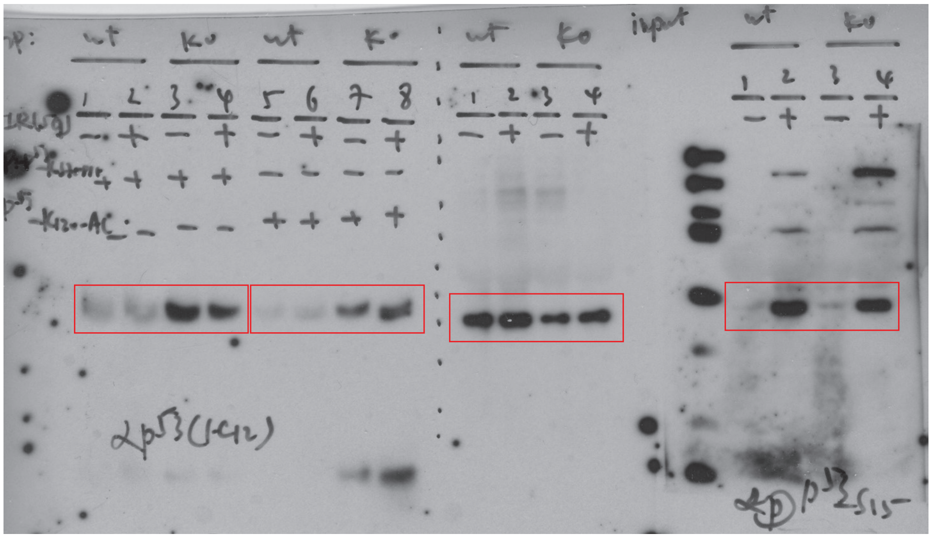

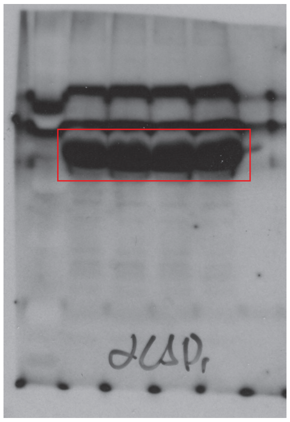

p53 (IP: p53-me2K370) | p53 (IP: p53-acK117) | p53 Input | p53 pS15 | LSD1

Figure 2B

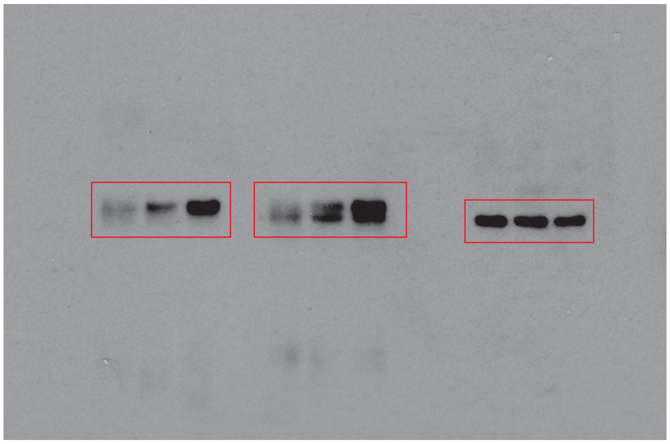

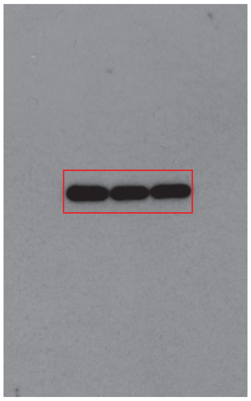

p53 (IP: p53-me2K370) | p53 (IP: p53-meK372) | p53 (Input) | LaminB1 (Input)

Figure 2C

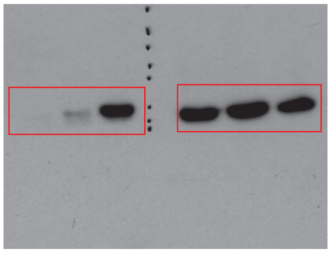

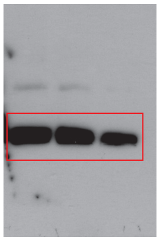

p53 (IP) | p53 (Input) | LSD1

Figure 2D

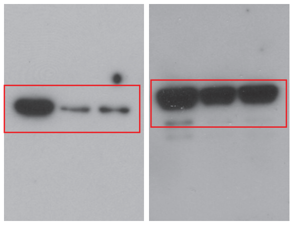

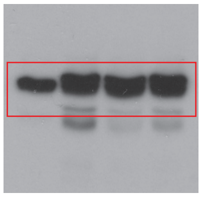

p53-me2K370 | p53 | p53 (Input)

Supplementary Figure 4
Uncropped images of blots shown in Figure 2.

Figure 2E

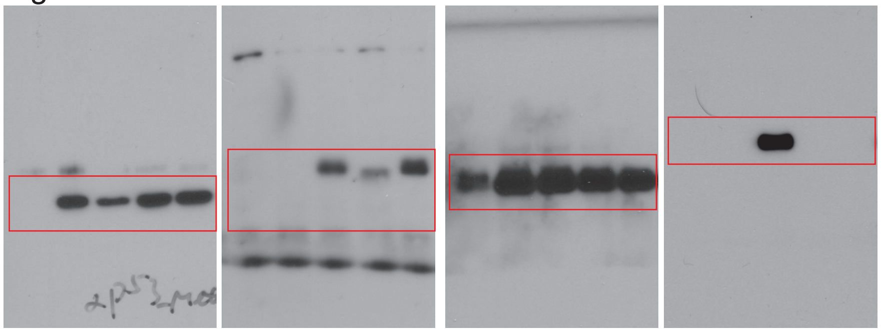

p53-me2K370 | Gfi1 | p53 | LSD1

Figure 3C

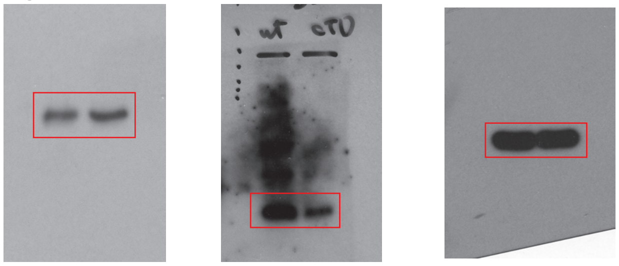

p53 (IP: p53-acK117) | p53 (input) | LaminB1

Figure 3G

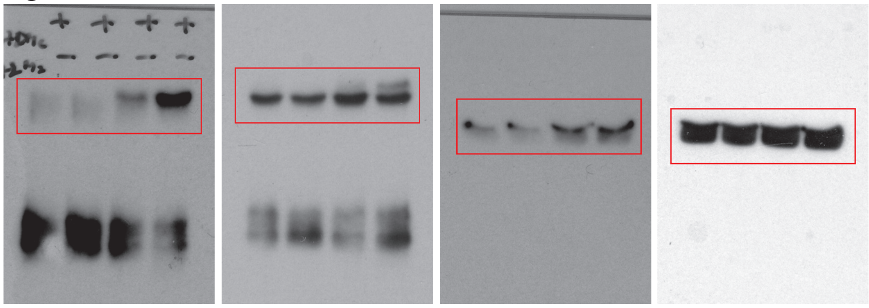

p53 (IP: p53-me2K370) | p53 (IP: p53-me2K372) | p53 (Input) | Actin

Supplementary Figure 1A

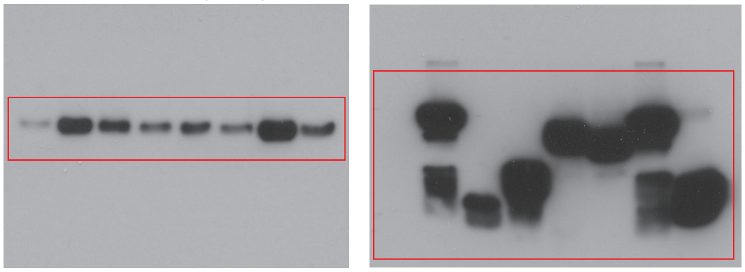

p53 | Flag

Supplementary Figure 1B

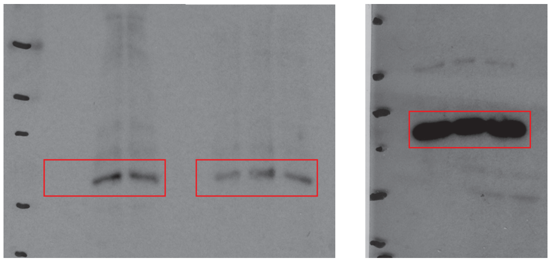

p53 (IP: GFI1) | p53 (input) | LaminB1 (input)

Supplementary Figure 2B

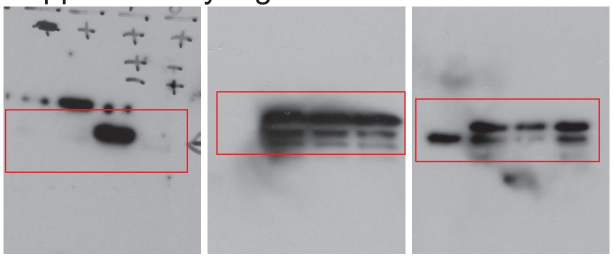

p53-me2K370 | p53 | p53 (Input)

Supplementary Figure 2C

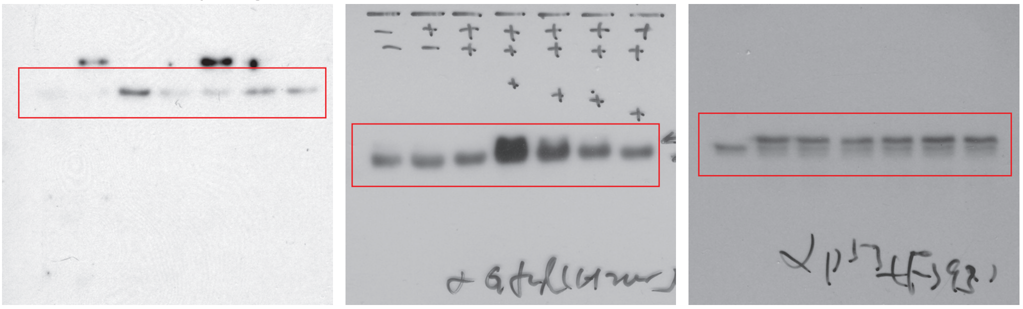

p53-me2K370 | Gfi1 | p53 (Input)

Supplementary Figure 5

Uncropped images of blots shown in Figure 3 and Supplementary Figures 1 and 2.
